# Supplementary material for: Nanoscale Roughness and Morphology Affect the IsoElectric Point of Titania Surfaces
Source: PLoS One. 2013 Jul 16;8(7):e68655. doi: 10.1371/journal.pone.0068655 (PMC3712945; doi:10.1371/journal.pone.0068655)
Supplement: Methods S1 — Characterization of surface morphology by Atomic Force Microscopy; characterization of colloidal probe radius; details on force curves and curve fitting procedures; applicability of the constant charge model for DLVO force; bibliography. (PDF) [file pone.0068655.s001.pdf]

Nanoscale roughness and morphology affect the IsoElectric Point of titania surfaces

F. Borghi[1], V. Vyas[1,2,†], A. Podestà[1]*, P. Milani[1]

1) Interdisciplinary Centre for Nanostructured Materials and Interfaces (C.I.Ma.I.Na.) and Dept. of Physics, Università degli Studi di Milano, Milano, Italy.

2) European School of Molecular Medicine (SEMM), IFOM-IEO Campus, Milano, Italy.

† Present address: Institute of Material Sciences, University of Connecticut, Storrs CT, United States.

* Corresponding author. E-mail: alessandro.podesta@mi.infn.it

SUPPORTING METHODS S1

Table of Contents

1. Characterization of surface morphology by Atomic Force Microscopy 2
2. Characterization of colloidal probe radius 3
3. Details on force curves and curve fitting procedures 5
   3.1. Applicability of the constant charge model for DLVO force 8
   3.2. Fitting strategy 8
4. Bibliography 10

1. Characterization of surface morphology by Atomic Force Microscopy

AFM images were processed using custom routines written in a Matlab environment. The RMS roughness (R_q) is calculated as $Rq = \sqrt{\frac{1}{N}\sum_{i,j}(h_{ij} - \bar{h})^2}$, where h_{ij} are height values in the topographic map (i,j are the row and column indices) and N is the number of pixels in the map, $\bar{h}$ is the average height ($\bar{h} = \frac{1}{N}\sum_{i,j} h_{ij}$). The specific area A_{spec} is the ratio of the three-dimensional area calculated on the image to the projected area, i.e. to the AFM scanning area. It is calculated as $A_{spec} = \frac{1}{N}\sqrt{1 + |\nabla h_{ij}|^2}$, where $|\nabla h_{ij}|$ is the modulus of the discretized surface gradient.

The specific area calculated from AFM images is always underestimated because of the inability of the AFM tip to detect overhangs and because of its finite size (typical AFM A_{spec} values do not exceed 2). The in-plane correlations of self- affine surfaces (or profiles) are described by two exponents: the Hurst exponent *H* and the correlation length ξ, which is the characteristic length over which two randomly chosen points on the surface (or on the profile) have uncorrelated heights. The average quadratic difference between heights of two points separated by a distance Δx (also called the height-height correlation function) scales as Δx^{2H} for $\Delta x < \xi$, then it saturates. An example is provided in Fig. S1 (here $\sigma \equiv R_q$ and C_2 is the h-h correlation function squared).

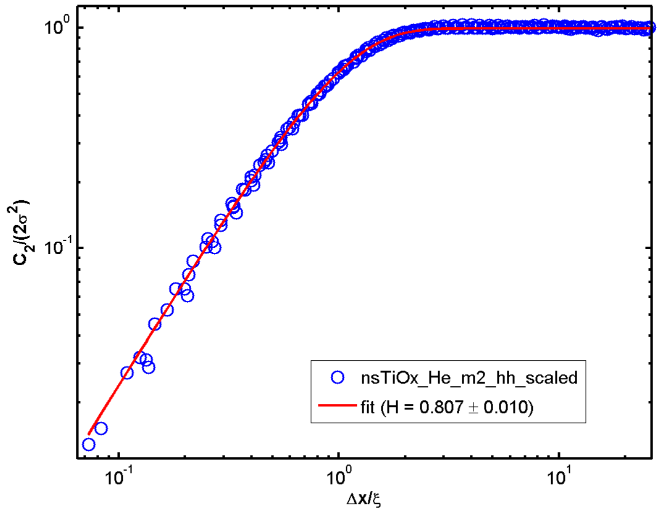

Figure S1. Initial linear region and saturation of the height-height correlation function.

The mesoscopic slope of the interface can be calculated as $2R_q/\xi$ (see Fig. 7A in the main text; this result is strictly valid only for a Gaussian surface [1]). For a surface with gaussian distribution of surface heights, the mesoscopic specific area can be calculated as $A_{spec} = 1+2(R_q/\xi)^2$ [1]. Being the

determination of both R_q and ξ reliable, the estimation of the mesoscopic specific area is such, as well; it has to be noted that this mesoscopic value fails in reproducing the gain in available area due to sub-correlation length surface structures. Table 1 in the main text reports the value of the punctual specific area calculated directly from AFM topographical maps as described above.

2. Characterization of colloidal probe radius

We have calibrated the radius of colloidal probes following a procedure recently introduced [2]. We have imaged a calibration grating array (MikroMasch TGT01) of sharp spikes with apical radius less than 10nm and tip angles below 25° with a cantilever equipped with the colloidal probe used in the DLVO experiments. The lateral and diagonal separations of the spikes are 2.12 and 3 μm, accordingly, and the height of the spikes is in the 600-800 nm range. Therefore, due to the very high aspect-ratio of reference sample features convolution between the geometry of the colloidal probe and the sample morphology is at its maximum, and the captured image (Fig. S2A) is the inverted image of the AFM probe, which can be modeled by spherical caps (Fig. S2B).

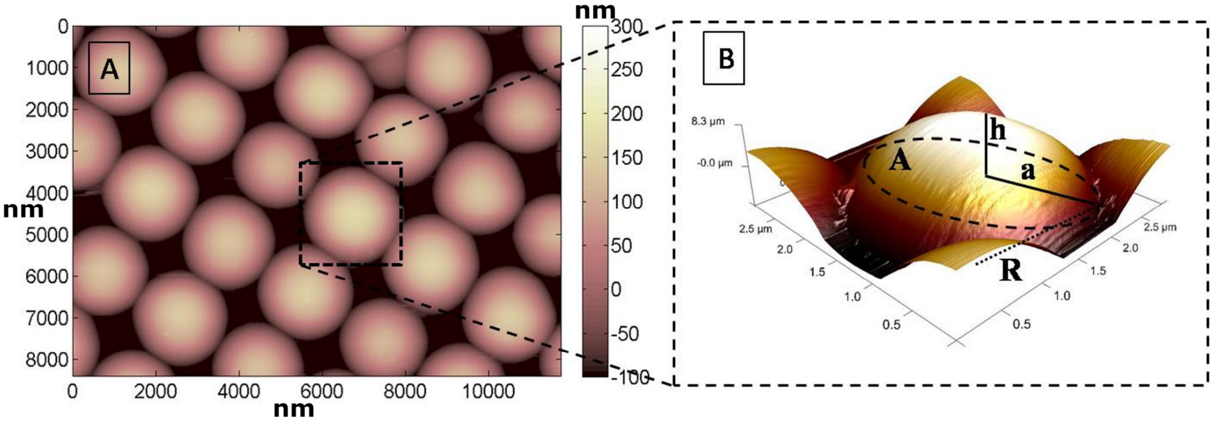

Figure S2. (A) AFM image of TGT01 grating obtained with a colloidal probe of nominal radius R=2.5 μm. Axes are in nm units. (B) 3D magnified view of an inverted AFM image of the probe and its geometrical parameters: the base radius a and area $A=\pi a^2$, the height h of the spherical cap and the radius of the mother sphere R.

The value of the probe radius and its error are extracted applying a statistical analysis of AFM topographs. In Fig. S3 are shown the Volume vs Height data of the spheres and the estimation of the radius extracted from the fit to the equation $V = \pi/3\ h^2\ (3R - h)$, V and h being the measured volume and height of the spherical caps found in AFM topographic maps like the one shown in Fig.

S2A (R_{best}= 2170 ± 65 nm). The microsphere has been attached to a rectangular tipless silicon cantilever (Nanosensors) and the force constant, determined by thermal tuning [3], is 0.43 N/m.

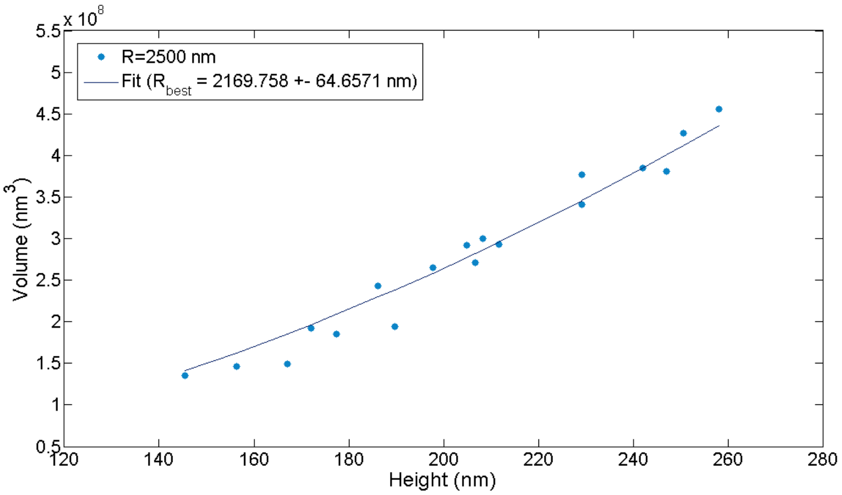

Figure S3. Volume versus height data extracted by the inverted AFM images of the colloidal probe used in the DLVO experiments on the TGT01 calibration sample.

3. Details on force curves and curve fitting procedures

Typical average force curves, acquired using a borosilicate glass colloidal probe with radius R=2170±65 nm on a flat glass borosilicate surface with [NaCl] varying in the range 0.1-100 mM and fixed neutral pH (≈6.5), are shown in Fig. S4.

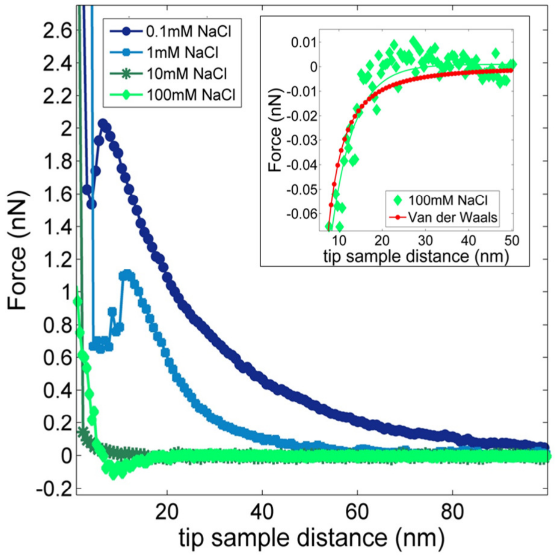

Figure S4. Average force curves between borosilicate glass colloidal probe and a flat borosilicate glass coverslip, acquired in solution with different ionic strength (0.1mM – 100mM NaCl). In the inset it is shown the overlapping between Van der Waals force curve (calculated using $A=0.8*10^{-20}$ J) and the experimental curves in 100mM NaCl solution.

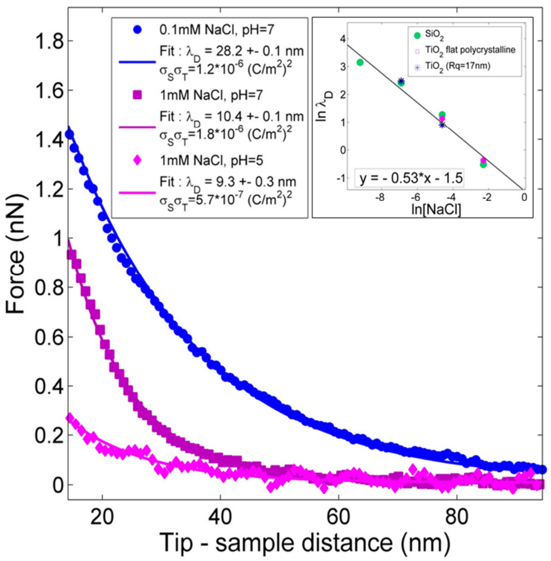

Figure S5. Best fit of average force curves between borosilicate glass colloidal probe and a flat borosilicate glass coverslip, acquired in solution with different ionic strength (0.1mM and 1mM NaCl, pH=7) or with the same ionic strength but different pH (1mM NaCl, pH_1=7 and pH_2 =5). In the inset, log plot of the Debye length versus the inverse of the square root of NaCl concentration, calculated in experiments with different substrates.

Error bars on average force data (not shown here, see main text) are calculated summing in quadrature two errors: a statistical error, typically negligible, calculated as the standard deviation of the mean of force values that are averaged, and a systematic error due to the calibration of the AFM cantilever, which is determined considering a 2% error due to deflection sensitivity calibration (see Mats&Methods in the main text) and 5% error due to the force constant calibration. Interpretation of force curves is the following. The tip, approaching the surface, remains in its rest position (constant deflection signal) until at a certain distance from the surface, depending on the ionic strength of the solution, it feels first the long-range electrostatic interaction with the sample surface and subsequently the Van der Waals attraction force [4,5]. An increased salt concentration (or an

increased Ionic strength of the solution) determines a decrease of the electrostatic force, even if the repulsion grows steeper. At the same time, the jump-in due to the Van der Waals attraction, takes place at larger distance from the surface; by increasing the salt concentration it shifts from 7 to 18nm. The smearing of the force curves at short distances is an artifact caused by the averaging process, due to the fact that the jump-in distance fluctuates by several nm from curve to curve; DLVO fit is performed in the large-distance region, typically between 10 and 100 nm, well before the onset of the jump-in. At the highest salt concentration the electrostatic repulsion is completely overwhelmed by Van der Waals attraction; a minimum appears, due to van der Waals force, while only at the shortest distance electrostatic repulsion can be appreciated. An expanded view of this curve is shown in the inset of Fig. S4, together with the Van der Waals contribution evaluated by the second term of Eq. 6 (main text) using $A=0.8\ 10^{-20}$ J.

It is very important to control the pH of the solution before and after AFM measurements in order to check the stability of the system and guarantee the accuracy in the determination of the IEP. It is also important to wait more than fifteen minutes after the immersion of the thin film and tip in the solution and to rinse the surfaces, before and after measurements, with neutral distilled water, in order to reach the equilibrium stability and to restore surface charges. Experimental data confirm that different ionic strengths determine the value of the Debye length according to Eqs. 3,4 without affecting the $\sigma_S\sigma_T$ value, while for the same value of Ionic Strength, $\sigma_S\sigma_T$ decreases with the pH of the solution until the value equals the first IEP of the system. Representative force curves and their best fit (using Eq.6) are shown in Fig. S5. The inset of Fig. S5 shows experimental values of λ_D measured in different salt concentrations solution, with different surfaces (SiO_2, flat polycrystalline TiO_2 and rough ns-TiO_2). λ_D scales as the inverse of the square root of $[NaCl]^{-1/2}$, as predicted by Eq. 4. We have also verified the stability of the solutions characterized by different value of pH during a period of one month, in the pH range between 3 and 7. pH values were checked using a pH meter. We have chosen to fix the 1mM NaCl concentration because it allows us to analyze a large range of pH values without changing Ionic strength of the solution and also because, in a more concentrated solution, the 1:1 electrolyte is no more completely inert for SiO_2 and TiO_2, promoting a shift of the IEP. Furthermore, for 1mM NaCl solution, the Debye length ($\lambda_D \sim 9.6$ nm) is large enough to guarantee a wide interval of electrostatic interaction and a higher signal-to-noise ratio.

**Borosilicate glass probe vs borosilicate glass substrate**	**Borosilicate glass probe vs rutile 100 TiO_2**	**Borosilicate glass probe vs ns-TiO_2 (pH=5.4)**
pH=3.4 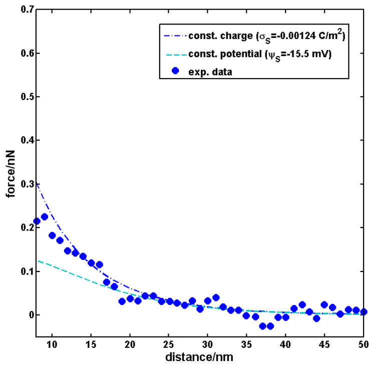	pH=4.28 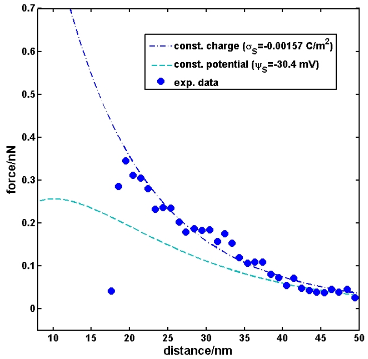	Rq=14 nm 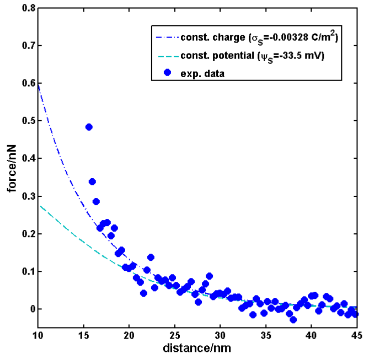
pH=5.25 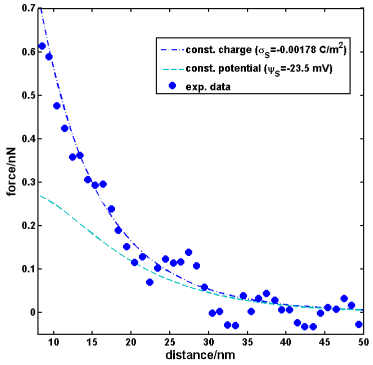	pH=5.4 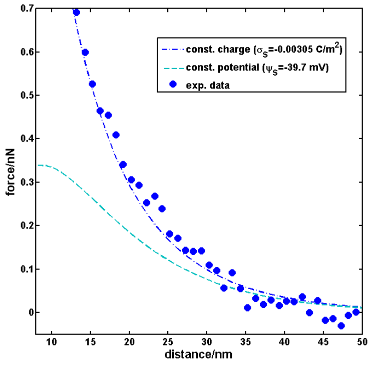	Rq=22 nm 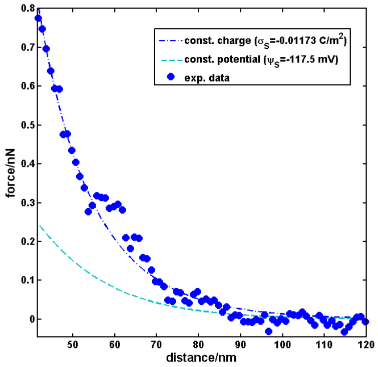
pH=6.8 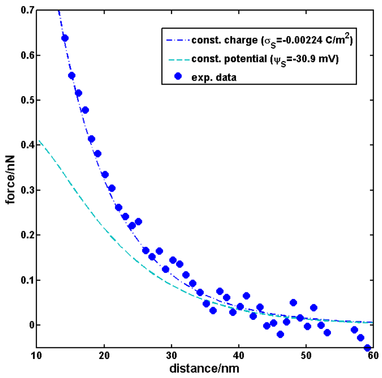	pH=7.9 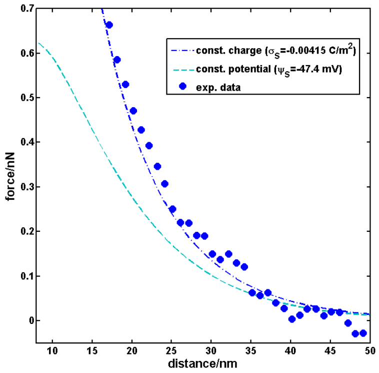	Rq=26nm 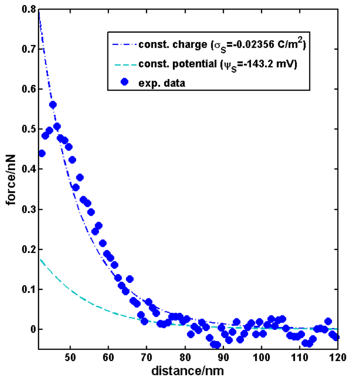

Figure S6. Comparison of force data acquired using a borosilicate glass colloidal probe on borosilicate glass substrate, rutile <100> and nanostructured TiO_2, with constant charge and constant potential curve obtained from nonlinear regression via Eq. 1 and from Eq. 2, using potentials calculated by Grahame equation (Eq. 5).

3.1. Applicability of the constant charge model for DLVO force

We have tested the applicability of the constant charge DLVO force model (Eq. 1 in the text, and its approximation for larger distances, Eq. 6), which is typically found to describe appropriately DLVO interactions between insulating oxide surfaces in aqueous electrolytes. Both constant charge and constant potential models (Eq. 1 and Eq. 2) overlap at distances sufficiently larger than λ_D, where Eqs. 1,2 reduce to a single exponential term whose prefactor contains the product of surface charges or surface potential, depending on the boundary conditions; charge densities and potentials are related by Grahame equation (Eq. 5). We have fitted the force curves by Eq. 1 across a distance range exceeding $1.5\lambda_D$, and used Grahame equation to calculate the diffuse layer potentials from the values of the diffuse charge densities σ_S and σ_T (the AFM probe-borosilicate glass substrate system was considered symmetric, which allowed to determine the absolute charge density of the probe; the latter parameter was kept fixed in fitting curves of other systems). It turned out that constant potential force curves systematically underestimate experimental data (Fig. S6), while the constant charge model could fit data across the complete range of distances (from jumpin to about 50 nm).

3.2. Fitting strategy

Eq. 1 and Eq. 6 overlap at sufficiently large distances; by fitting the force curves data with Eq.6 at distances larger than approximately $1.5\lambda_D$ it was possible to determine the Debye length and the product $\sigma_S\sigma_T$ of charge densities. If one knows the charge density of one of the two surfaces, the other can be determined. In particular, on symmetric systems $\sigma_S \approx \sigma_T$ and therefore $\sigma_T \approx \sqrt{\sigma_S\sigma_T}$. We could therefore characterized the net surface charge density of the colloidal probe from force measurements in aqueous electrolyte on a borosilicate glass substrate (see section 2.1 of file Supporting Text S1); we have then used the values of σ_T at different pH to calculate the absolute net charge density σ_S of crystalline and nanostructured TiO_2 surfaces.

For each sample 100 force curves were typically acquired in six different locations (separated by 100μm) in order to accurately characterize the Debye length and the charge densities of the surfaces. Charge densities and Debye lengths extracted from average force curves of different locations were averaged; their errors were estimated as the 68% confidence interval according to the optimized strategy discussed by Lybanon [6], consisting in repeating the fit on a set of artificial experimental data obtained by summing a Gaussian error to the original data based on errors on both force and distances, then looking at the dispersion of fit parameters obtained. For both Debye

lengths and charge densities, the error δ associated to the averages across different locations was calculated propagating the errors δ_i of the nonlinear regression through the arithmetic mean function, i.e. $\delta = {}^{1}/_{N}\sqrt{\sum_i^N \delta_i^2}$.

4. Bibliography

1. Daikhin L I, Kornyshev A A, Urbakh M (1996) Double-layer capacitance on a rough metal surface. Physical Review E 53: 6192-6199. DOI: 10.1103/PhysRevE.53.6192.
2. Indrieri M, Podestà A, Bongiorno G, Marchesi D, Milani P (2011) Adhesive-free colloidal probes for nanoscale force measurements: production and characterization. Rev. Sci. Instrum. 82: 023708-10. DOI: 10.1063/1.3553499.
3. Butt H, Jaschke M (1995) Calculation of thermal noise in atomic force microscopy. Nanotechnology 6: 1-7. DOI: 10.1088/0957-4484/6/1/001.
4. Butt H J, Cappella B, Kappl M (2005) Force measurements with the atomic force microscope: Technique, interpretation and applications. Surface Science Reports 59: 1–152. DOI: 10.1016/j.surfrep.2005.08.003.
5. Leckband D, Israelachvili J (2001) Intermolecular forces in biology. Quarterly Reviews of Biophysics 34: 105-267. DOI: 10.1017/S0033583501003687.
6. Lybanon M (1984) A better least-squares method when both variables have uncertainties. Am. J. Phys. 52: 22-26.
