# Supplementary material for: Nanoscale Roughness and Morphology Affect the IsoElectric Point of Titania Surfaces
Source: PLoS One. 2013 Jul 16;8(7):e68655. doi: 10.1371/journal.pone.0068655 (PMC3712945; doi:10.1371/journal.pone.0068655)
Supplement: Text S1 — Charging of surfaces in liquid electrolytes; determination of charge density products and IEPs of reference systems; self-overlap of electrostatic double-layers: a simplified picture; bibliography. (PDF) [file pone.0068655.s002.pdf]

Nanoscale roughness and morphology affect the IsoElectric Point of titania surfaces

F. Borghi[1], V. Vyas[1,2,†], A. Podestà[1]*, P. Milani[1]

1) Interdisciplinary Centre for Nanostructured Materials and Interfaces (C.I.Ma.I.Na.) and Dept. of Physics, Università degli Studi di Milano, Milano, Italy.

2) European School of Molecular Medicine (SEMM), IFOM-IEO Campus, Milano, Italy.

† Present address: Institute of Material Sciences, University of Connecticut, Storrs CT, United States.

* Corresponding author. E-mail: alessandro.podesta@mi.infn.it

SUPPORTING TEXT S1

Table of Contents

1. Charging of surfaces in liquid electrolytes 2
2. Determination of charge density products and IEPs of reference systems 3
   2.1. Borosilicate glass colloidal probe and reference substrate 3
   2.2. Single crystal <100> and polycrystalline rutile TiO_2 5
   2.3. Nanostructured TiO_2 7
3. Self-overlap of electrostatic double-layers: a simplified picture 12
4. Bibliography 16

1. **Charging of surfaces in liquid electrolytes**

The charging behaviour of metal oxide surfaces in aqueous electrolytes is generally attributed to amphoteric character of surface hydroxyl groups [1-6]. Charging of the solid surface can be formally regarded as a two-step protonation of surface M-O$^-$ groups:

$$M\text{-}O^- + H^+ \leftrightarrow M\text{-}OH;\ K_1 \quad \text{(S1a)}$$

$$M\text{-}OH + H^+ \leftrightarrow M\text{-}OH_2^+;\ K_2 \quad \text{(S2)}$$

or to the interaction of surface hydroxyls M-OH with OH$^-$ and H$^+$ ions, in which case the first reaction must be replaced with:

$$M\text{-}OH + OH^- \leftrightarrow M\text{-}O^- + H_2O;\ K_1' \quad \text{(S1b)}$$

The equilibrium constants K_1 and K_2 are defined as: K_1=[M-OH]/([M-O$^-$][H$^+$]) and K_2=[M-OH$_2^+$]/([M-OH][H$^+$]), [X] representing the molar concentration of the species X. It turns out that $1/K_1'=K_wK_1$, $K_w=10^{-14}$ being the equilibrium constant of the dissociation reaction of water into H$^+$ and OH$^-$ ions (due to its very small value, pK_1 and pK_1' are almost equal, being $pK=-\log_{10}(K)$).

In addition to association/dissociation of surface hydroxyls described by Eqs. S1,S2, also adsorption of anions A$^-$ and cations C$^+$ from solution to charged surface sites may take place, according to reactions:

$$M\text{-}O^- + C^+ \leftrightarrow M\text{-}O^-\cdot C^+ \quad \text{(S3)}$$

$$M\text{-}OH_2^+ + A^- \leftrightarrow M\text{-}OH_2^+\cdot A^- \quad \text{(S4)}$$

where K_+=[M-O$^-\cdot$C$^+$]/[M-O$^-$][C$^+$] and K_-=[M-OH$_2^+\cdot$A$^-$]/[M-OH$_2^+$][A$^-$].

The surface charge density σ_0, the charge density at the inner Helmholtz plane σ_I, and the charge density of the diffuse layer at the outer Helmholtz plane σ_d are equal to [6]:

$$\sigma_0 = F\,([M\text{-}OH_2^+] + [M\text{-}OH_2^+\cdot A^-] - [M\text{-}O^-] - [M\text{-}O^-\cdot C^+])$$

$$\sigma_i = F\,([M\text{-}O^-\cdot C^+] - [M\text{-}OH_2^+\cdot A^-])$$

$$\sigma_d = -(\sigma_0+\sigma_i) = -F([M\text{-}OH_2^+] - [M\text{-}O^-])$$

where F is the Faraday constant, i.e. the number of coulombs per mole of electrons.

2. Determination of charge density products and IEPs of reference systems

Force curves have been acquired at 20°C in 1mM NaCl solutions at different pH (from 3 to 8), whose value is detected immediately before and after the AFM measurements by a pH meter.

We have fitted the average curves with Eq. 6, for distances larger than approximately 15-20 nm, and sufficiently far away from the jump-in point, in order to avoid the mix-up between electrostatic force and the repulsion in contact regime and to neglect the contribution of the term in Eq. 1 proportional to $exp(-2D/\lambda_D)$.

2.1. Borosilicate glass colloidal probe and reference substrate

In Fig. S7A electrostatic interactions at different pH between the colloidal borosilicate glass tip and the borosilicate glass coverslip are shown.

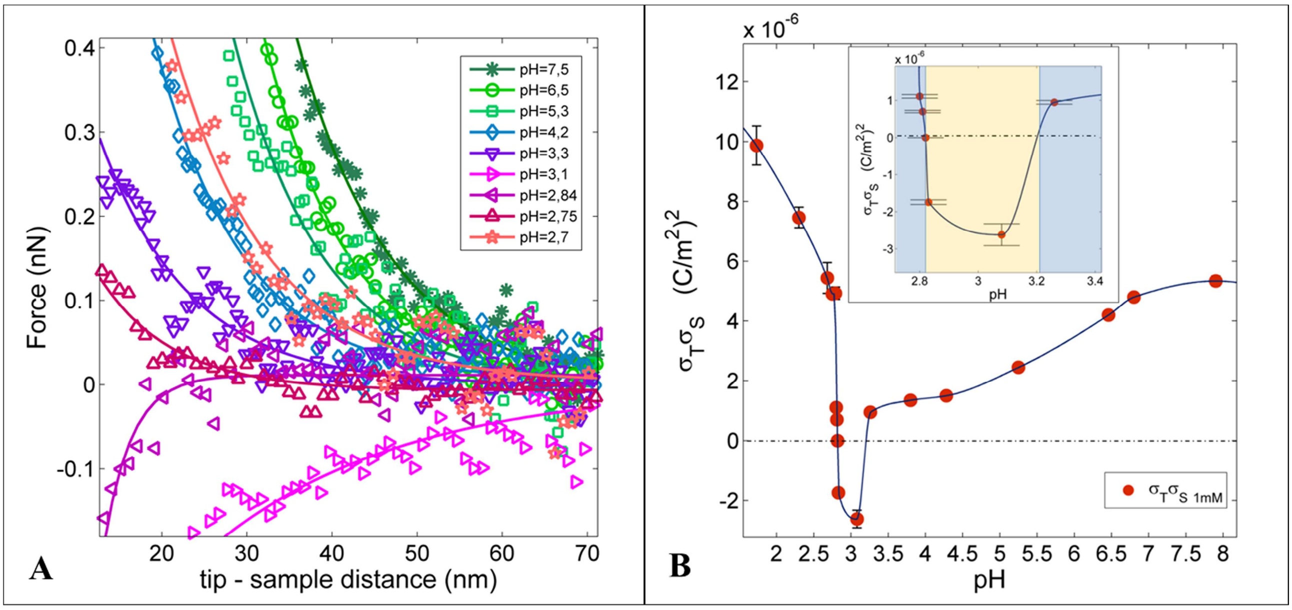

Figure S7 (A) Force curves in 1mM NaCl at different pH values between the colloidal borosilicate glass tip and the borosilicate glass coverslip and (B) the $\sigma_S\sigma_T$ versus pH, extracted from the best fit of force curves. In the inset a magnification that clearly shows the two IEP of the surfaces.

The decreasing repulsion with pH corresponds to a decrease of the double layer interaction. At pH=3.08 the double-layer interaction becomes attractive and the value of Van der Waals interaction (independent of pH) is negligible compared to it (at a tip-surface distance of 14nm they are -0.1 nN and -0.02 nN respectively). The shift from repulsive to attractive double-layer interaction indicates that the IEP of colloidal glass tip lies between pH 3.3 and 3.1. When the pH of the solution is far

enough from the IEP of the surface, the decay length is correctly described by the Debye Length of Eq. 4 (1mM NaCl at pH =3.81, λ_D= 9.49 nm).

In Fig. S7B is shown $\sigma_S\sigma_T$ extracted from the force curves as a function of pH and it is possible to identify immediately the IEP of the two surfaces. For high value of pH, both surfaces are negatively charged and so $\sigma_S\sigma_T$ is positive. Lowering the pH, we are approaching the first IEP of the system, and so the product of the surface charge density decreases until the zero value of the first surface IEP. When the pH value is lower than this first IEP value (pH_{IEP} = 3.2), the charge density sign of one surface of the system changes and the interaction becomes attractive. The product of surface charge densities remains negative until the second IEP of the system is reached (pH_{IEP}=2.8). The slopes of the two positive regions of $\sigma_S\sigma_T$ versus pH are not equal. In fact, at lower pH, we are adding 10^{-2} M HCl, while at higher value of pH the amount of HCl is order of magnitude lower and the slope of the charging curve grows very slowly [7]. Despite the fact that both the colloidal probe and the glass coverslip used in this study are made of borosilicate glass, we found evidence of an asymmetric interface characterized by two different pH_{IEP} values (Fig. S6B). The difference is small despite that fully resolved by our experimental apparatus (pH_{IEP}=3.20 ± 0.05 for the AFM probe vs pH_{IEP}= 2.82 ± 0.05 for the coverslip). The observed difference could be due to small changes in the relative abundances of silica and boron oxide components in borosilicate glasses, enhanced also by the different thermal annealing procedure and geometrical surface properties, which cause changes in the density of amphoteric sites (such compositional differences are in fact rather likely, due to batch-to-batch, as well as provider-to-provider fluctuations).

Assuming that the system is symmetric (a reasonable assumption due to the similarity of the IEPs of the probe and the substrate), and therefore $\sigma_T \approx \sqrt{\sigma_S \sigma_T}$, we have calculated the net surface charge density of the AFM colloidal probe (Fig. S8).

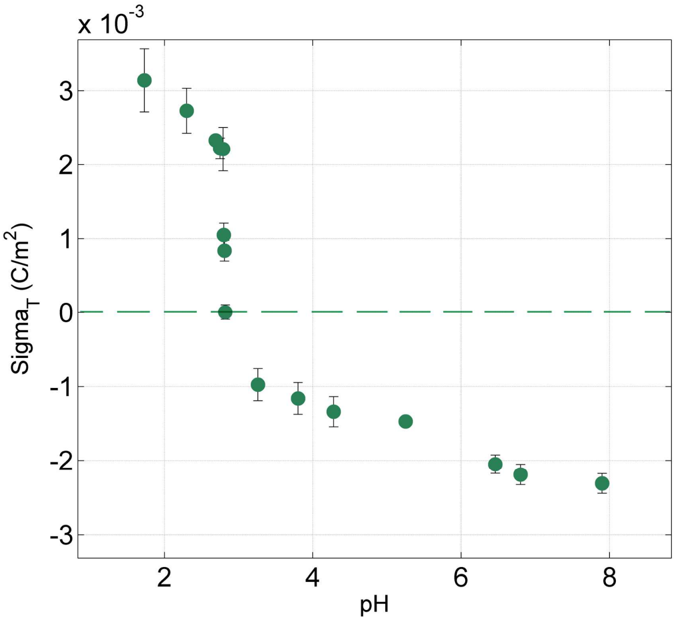

Figure S8. Net surface charge density of the AFM colloidal probe vs pH.

2.2. Single crystal <100> and polycrystalline rutile TiO_2

We have studied the interactions of the colloidal probe with reference single-crystal <100> and polycrystalline rutile TiO_2 surfaces (Table 2 in the main text). In the plots of $\sigma_S\sigma_T$ versus pH (Fig. S9B and S10B) it is possible to distinguish two different IEPs (one pertaining to the probe, the other to the sample). By comparing these plots, it is possible to determine precisely which one is the IEP of the probe; it recurred with high precision always in the same pH value for all the system studied, included the nanostructured ones (Fig. S11B-S19B). The attribution of pH_{IEP} =3.2 value to the AFM probe was supported by the observation that this value is systematically measured in all experiments (which share the same borosilicate colloidal probe).

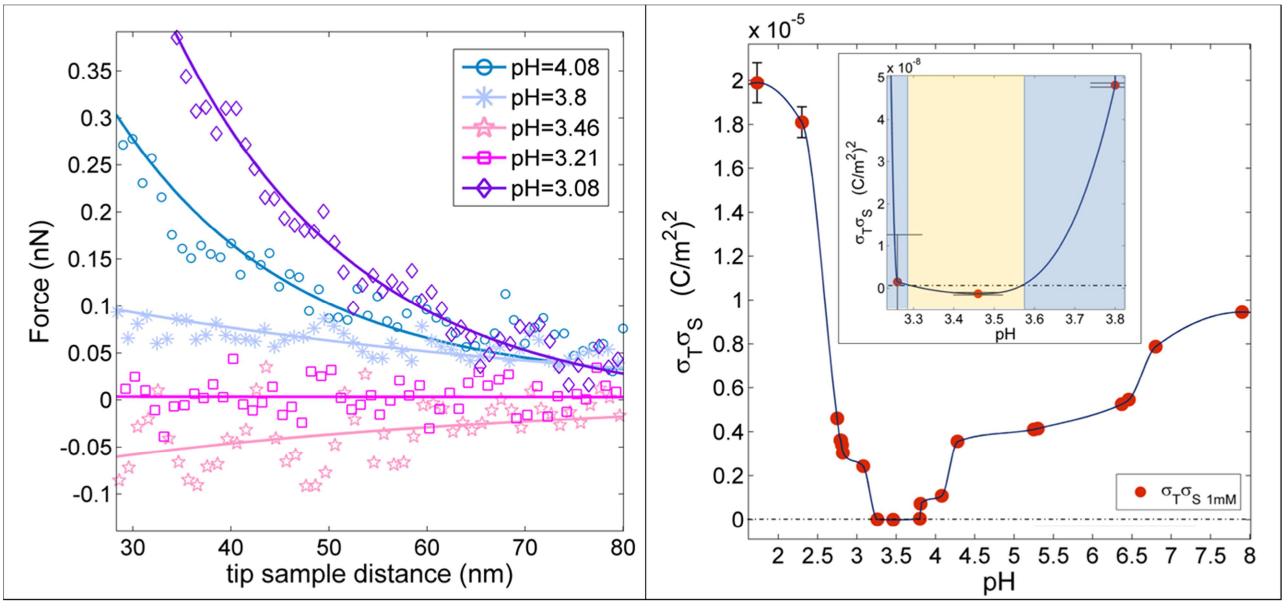

Figure S9. (Left) Force curves in 1mM NaCl at different pH values between the colloidal borosilicate glass tip and the Rutile flat TiO_2 substrate (crystallographic orientation <100>); (right) the $\sigma_S\sigma_T$ versus pH, extracted from the best fit of force curves. In the inset a magnification of the curve, which clearly identify the inversion of the charge sign due to the separation between the two IEPs of the system.

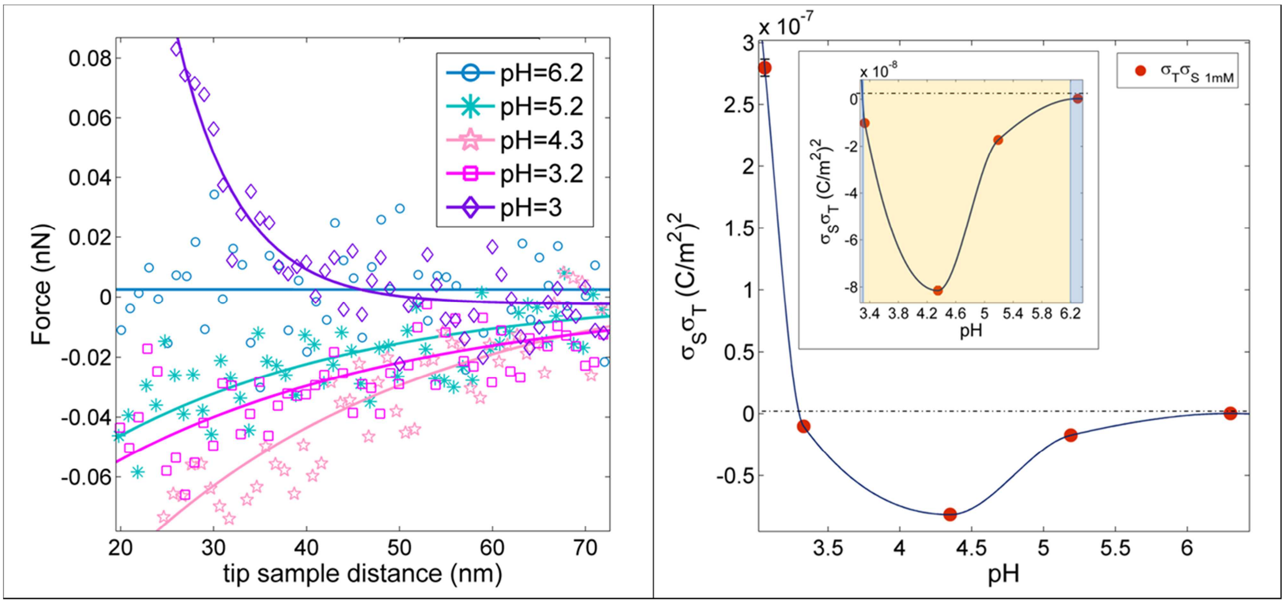

Figure S10. (Left) Force curves in 1mM NaCl at different pH values between the colloidal borosilicate glass tip and the Rutile flat polycrystalline TiO_2; (right) the $\sigma_S\sigma_T$ versus pH, extracted from the best fit of force curves. In the inset a magnification of the curve, which clearly identify the inversion of the charge sign due to the separation between the two IEPs of the system.

2.3. Nanostructured TiO_2

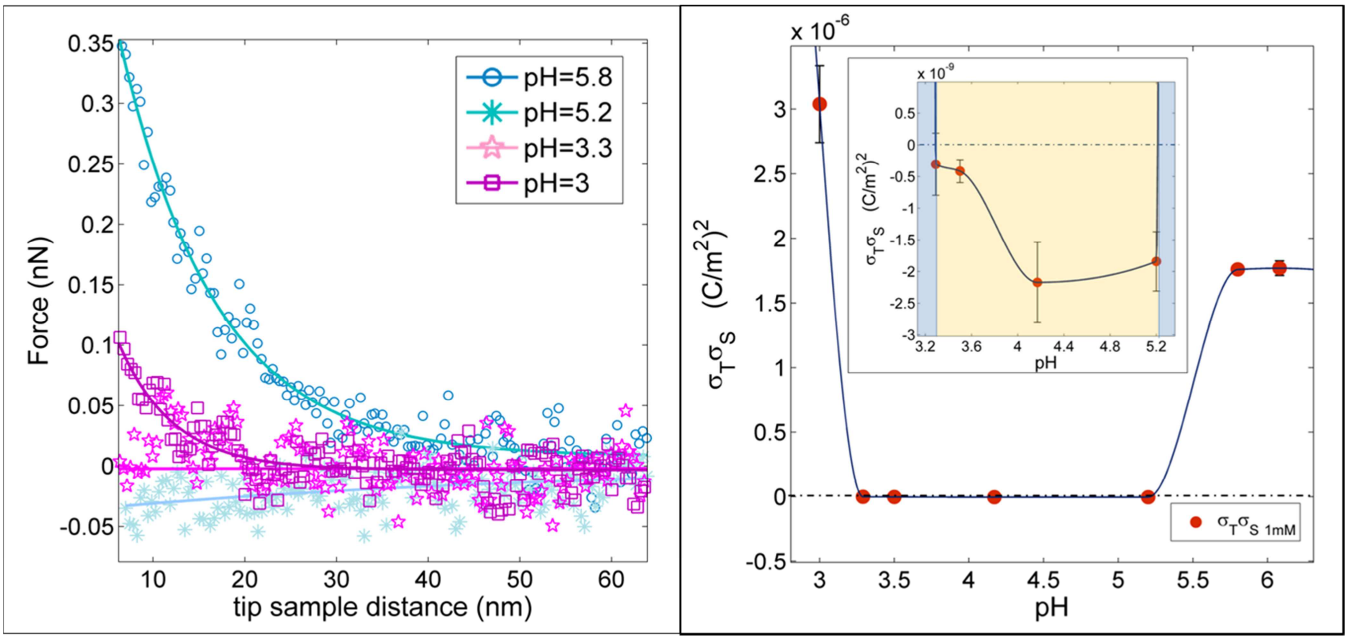

Figure S11. (Left) Force curves in 1mM NaCl at different pH values between the colloidal borosilicate glass tip and the rough ns-TiO_2 sample (Rq=5nm); (right) the $\sigma_S\sigma_T$ versus pH, extracted from the best fit of force curves. In the inset a magnification of the curve, which clearly identify the inversion of the charge sign due to the separation between the two IEPs of the system.

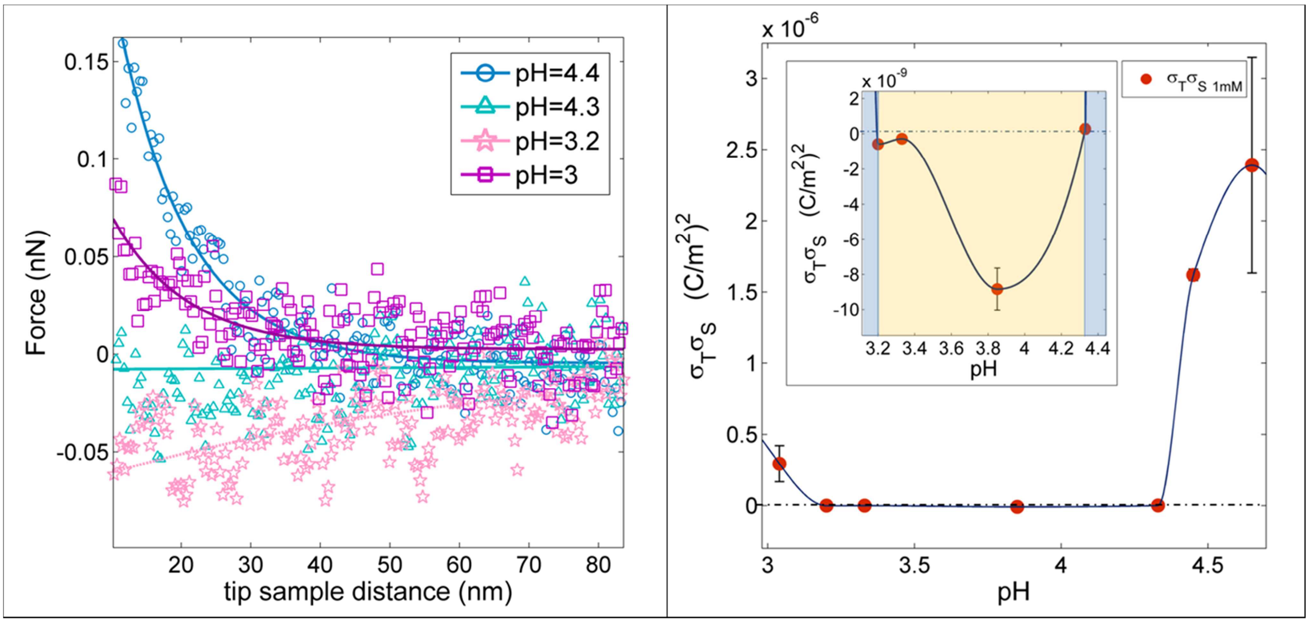

Figure S12. (Left) Force curves in 1mM NaCl at different pH values between the colloidal borosilicate glass tip and the rough ns-TiO_2 sample (Rq=10nm); (right) the $\sigma_S\sigma_T$ versus pH, extracted from the best fit of force curves. In the inset a magnification of the curve, which clearly identify the inversion of the charge sign due to the separation between the two IEPs of the system.

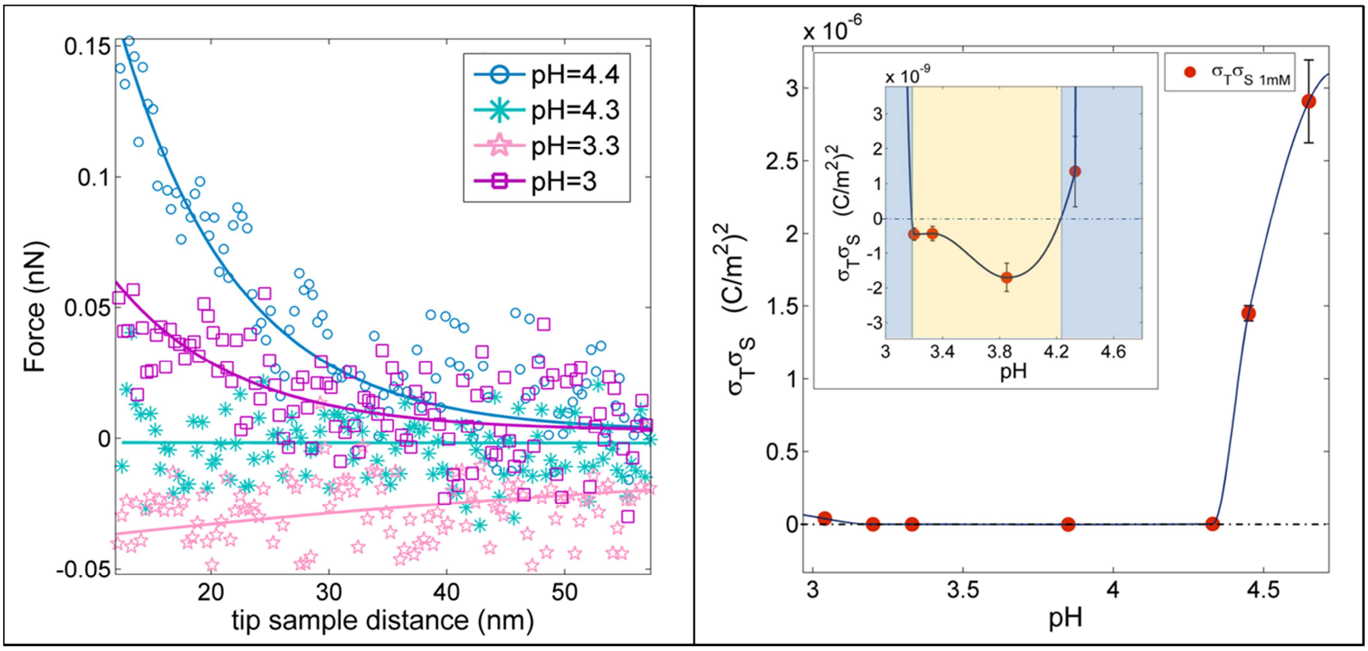

Figure S13. (Left) Force curves in 1mM NaCl at different pH values between the colloidal borosilicate glass tip and the rough ns-TiO_2 sample (Rq=14nm); (right) the $\sigma_S\sigma_T$ versus pH, extracted from the best fit of force curves. In the inset a magnification of the curve, which clearly identify the inversion of the charge sign due to the separation between the two IEPs of the system.

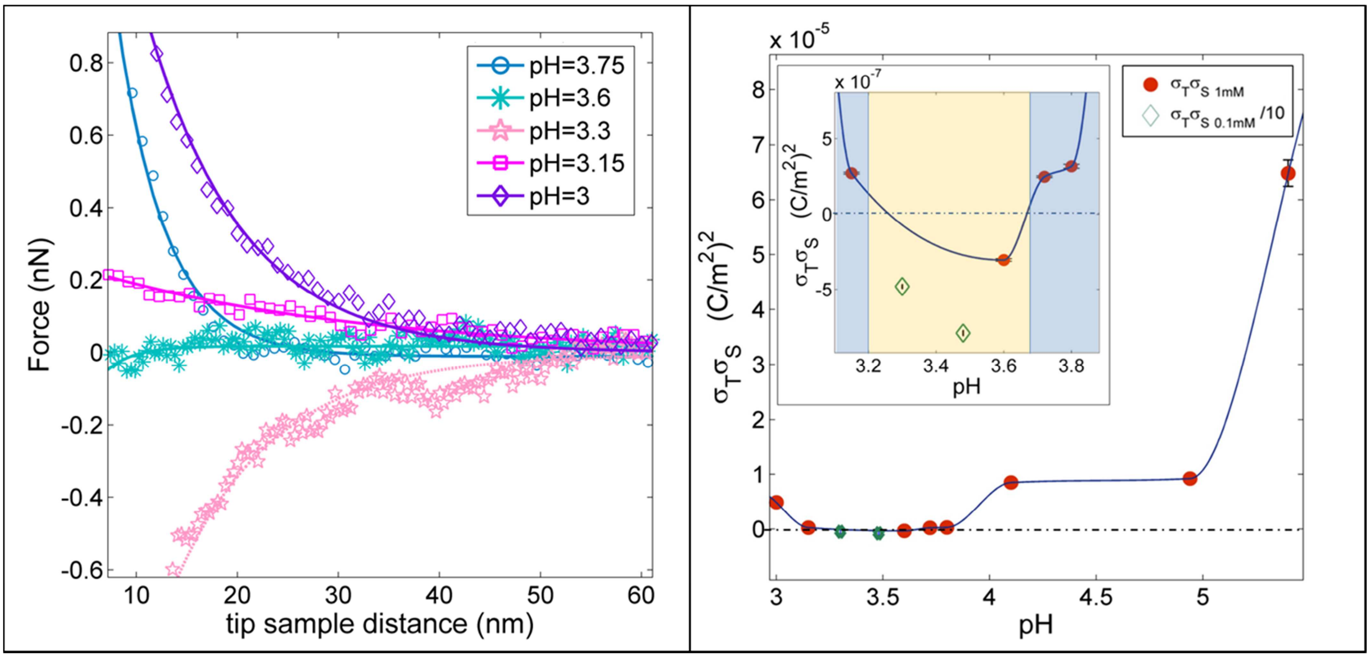

Figure S14. (Left) Force curves in 1mM NaCl at different pH values between the colloidal borosilicate glass tip and the rough ns-TiO_2 sample (Rq=17nm); (right) the $\sigma_S\sigma_T$ versus pH, extracted from the best fit of force curves. In the inset a magnification of the curve, which clearly identify the inversion of the charge sign due to the separation between the two IEPs of the system.

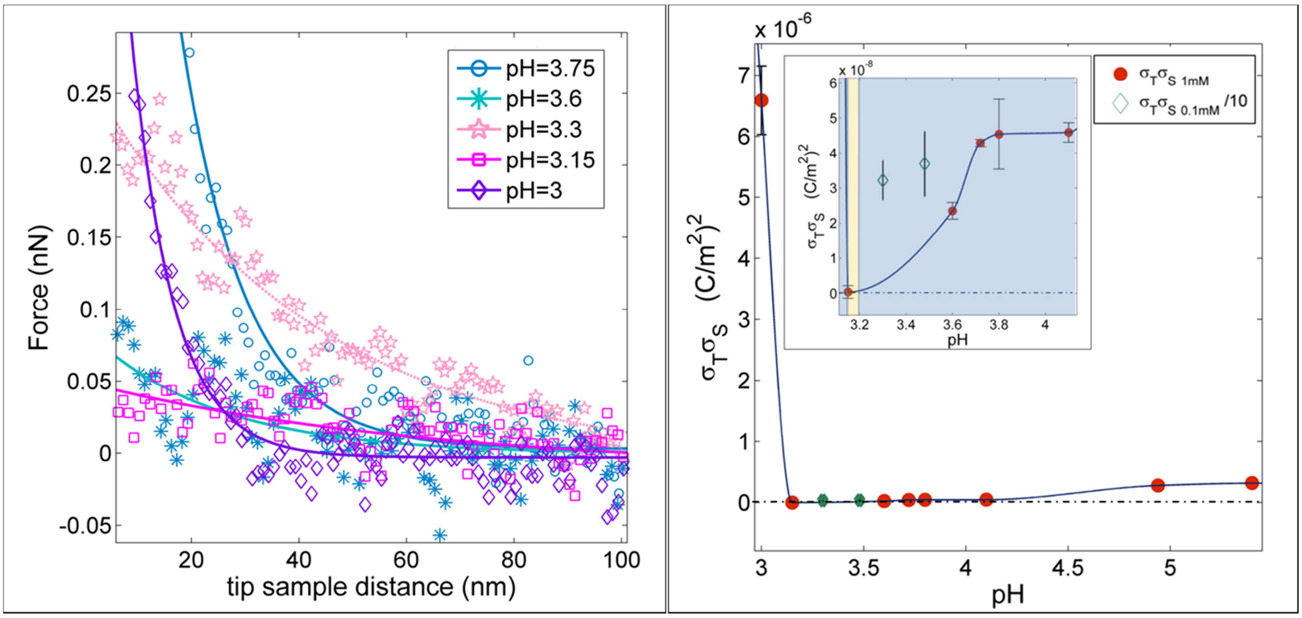

Figure S15. (Left) Force curves in 1mM NaCl at different pH values between the colloidal borosilicate glass tip and the rough ns-TiO_2 sample (Rq=19nm); (right) the $\sigma_S\sigma_T$ versus pH, extracted from the best fit of force curves. In the inset a magnification of the figure, which shows the overlapping between the two IEPs of the system.

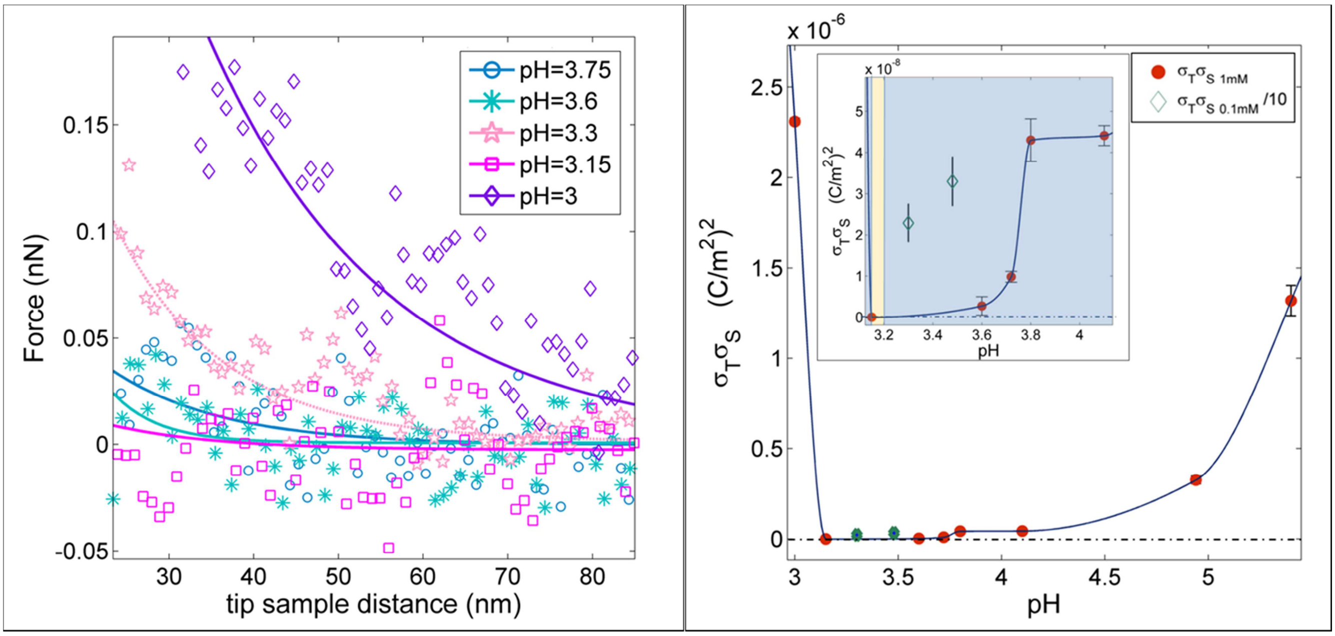

Figure S16. (Left) Force curves in 1mM NaCl at different pH values between the colloidal borosilicate glass tip and the rough ns-TiO_2 sample (Rq=20nm); (right) the $\sigma_S\sigma_T$ versus pH, extracted from the best fit of force curves. In the inset a magnification of the curve, which shows the overlapping between the two IEPs of the system.

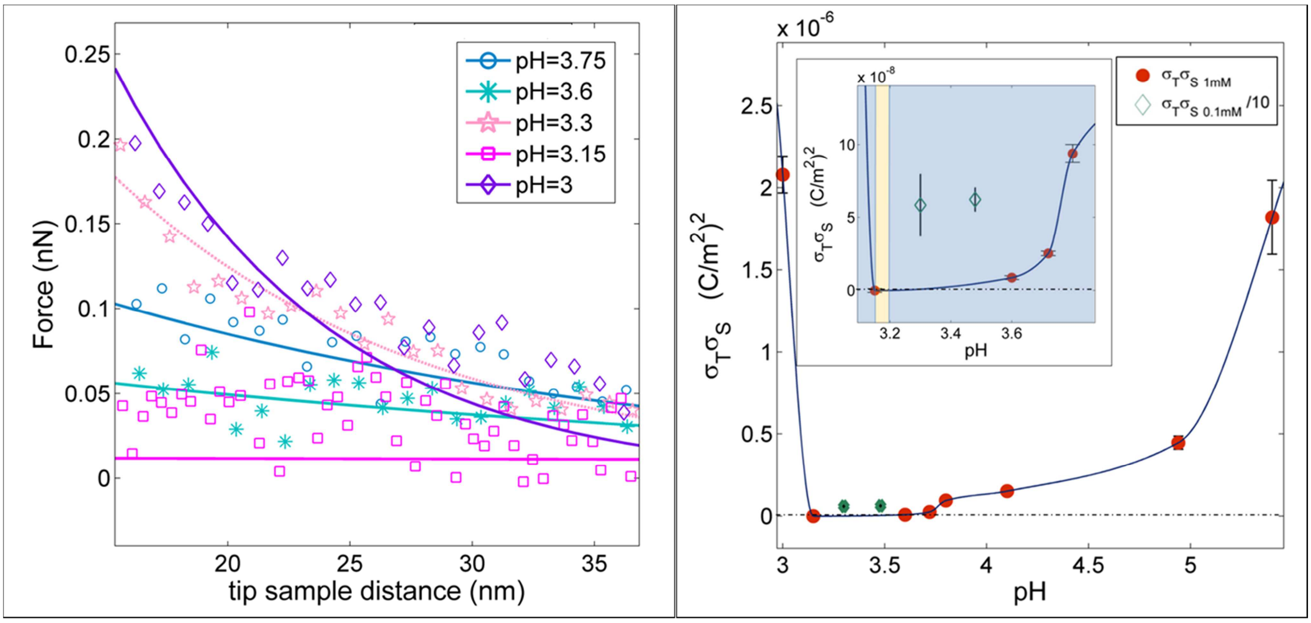

Figure S17. (Left) Force curves in 1mM NaCl at different pH values between the colloidal borosilicate glass tip and the rough ns-TiO_2 sample (Rq=21nm); (right) the $\sigma_S\sigma_T$ versus pH, extracted from the best fit of force curves. In the inset a magnification of the curve, which shows the overlapping between the two IEPs of the system.

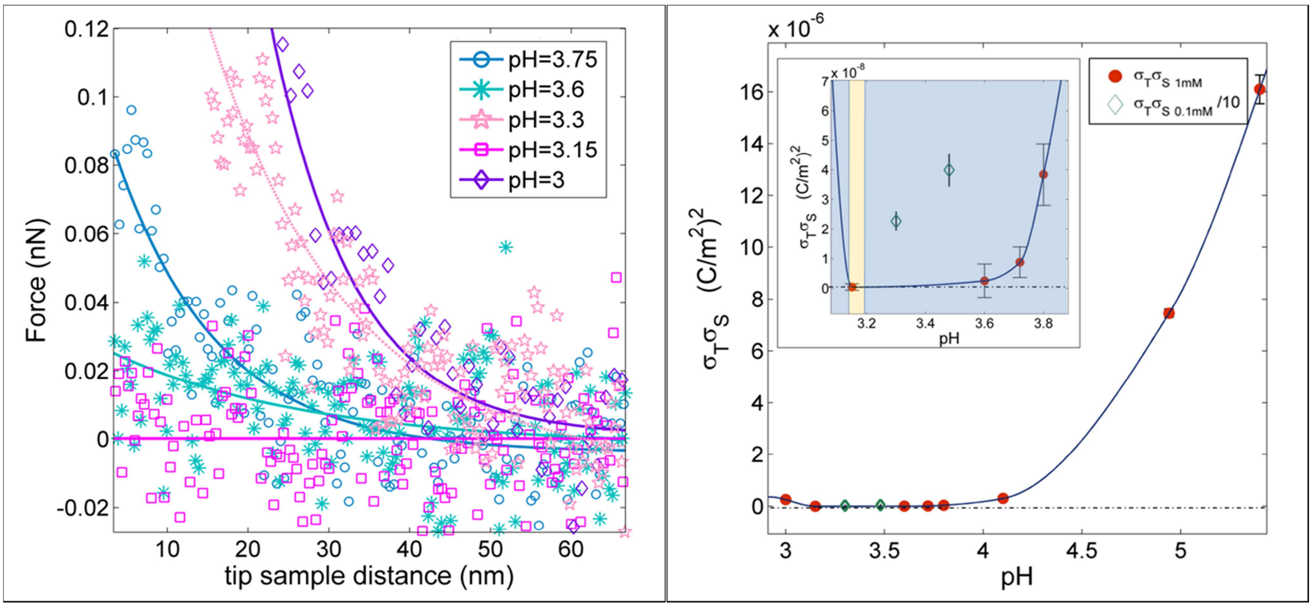

Figure S18. (Left) Force curves in 1mM NaCl at different pH values between the colloidal borosilicate glass tip and the rough ns-TiO_2 sample (Rq=22nm); (right) the $\sigma_S\sigma_T$ versus pH, extracted from the best fit of force curves. In the inset a magnification of the curve, which shows the overlapping between the two IEPs of the system.

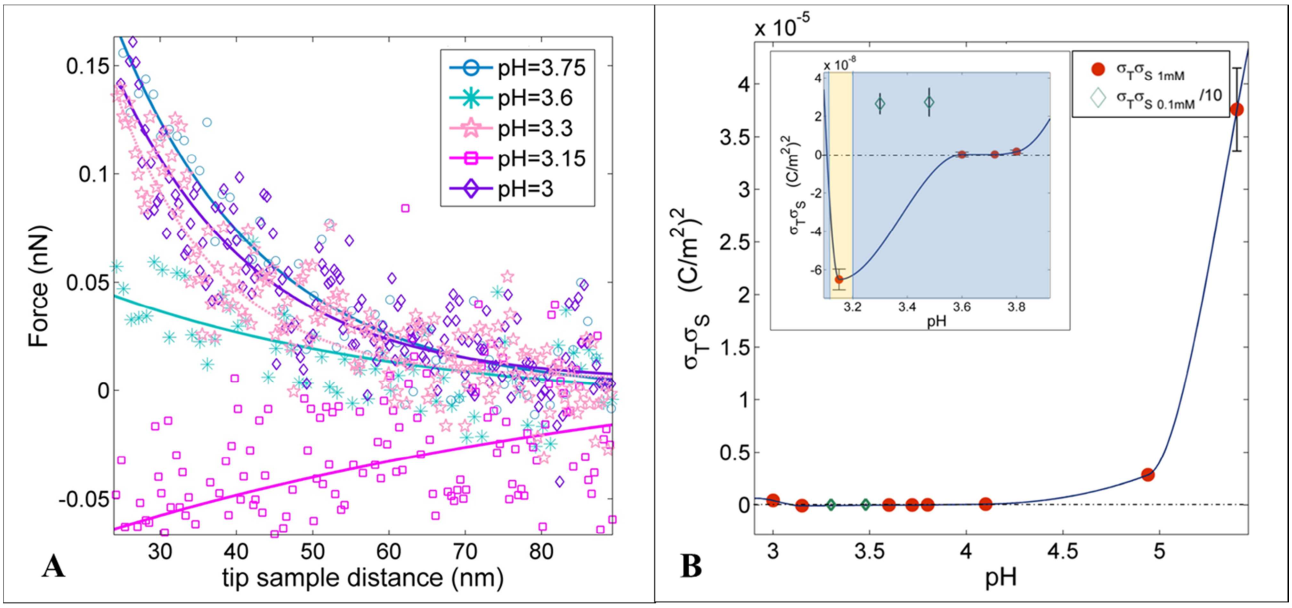

Figure S19. (Left) Force curves in 1mM NaCl at different pH values between the colloidal borosilicate glass tip and the rough ns-TiO_2 sample (Rq=26nm); (right) the $\sigma_S\sigma_T$ versus pH, extracted from the best fit of force curves. In the inset a magnification of the curve, which shows the overlap between the two IEPs of the system.

3. **Self-overlap of electrostatic double-layers: a simplified picture**

The double layer structure is assumed to consist in a volume of depth λ_D stemming perpendicularly from the solid surface toward the bulk of the electrolyte.

We consider as the total double layer volume of a single pore the sum of the two regions originating from the two slopes of the pore. We consider a 2-dimensional projection of the pore, so that the volume of the double layer is in fact an area Σ_0. Our results should be the same, apart from a multiplicative factor, in the 3-dim. case.

The pore has slope $\tan(\theta)=2R_q/\xi$ (see Fig. S20).

The area of the overlapping region is Σ. We introduce the self-overlap parameter $\gamma=\Sigma/\Sigma_0$.

We distinguish between two cases: $\theta\leq 45°$ ($2R_q/\xi\leq 1$), and $\theta>45°$ ($2R_q/\xi>1$). We will calculate the ratio γ only for $\lambda_D<\lambda^*_D$. λ^*_D represents the depth of the double layer at which the shape of the overlapping regin changes from a quadrilateral (a kite, for $\theta\leq 45°$, or a rhombus, for $\theta>45°$), from a more complex polygon. λ^*_D is shown for the two cases in Fig. S20-left and S21-left.

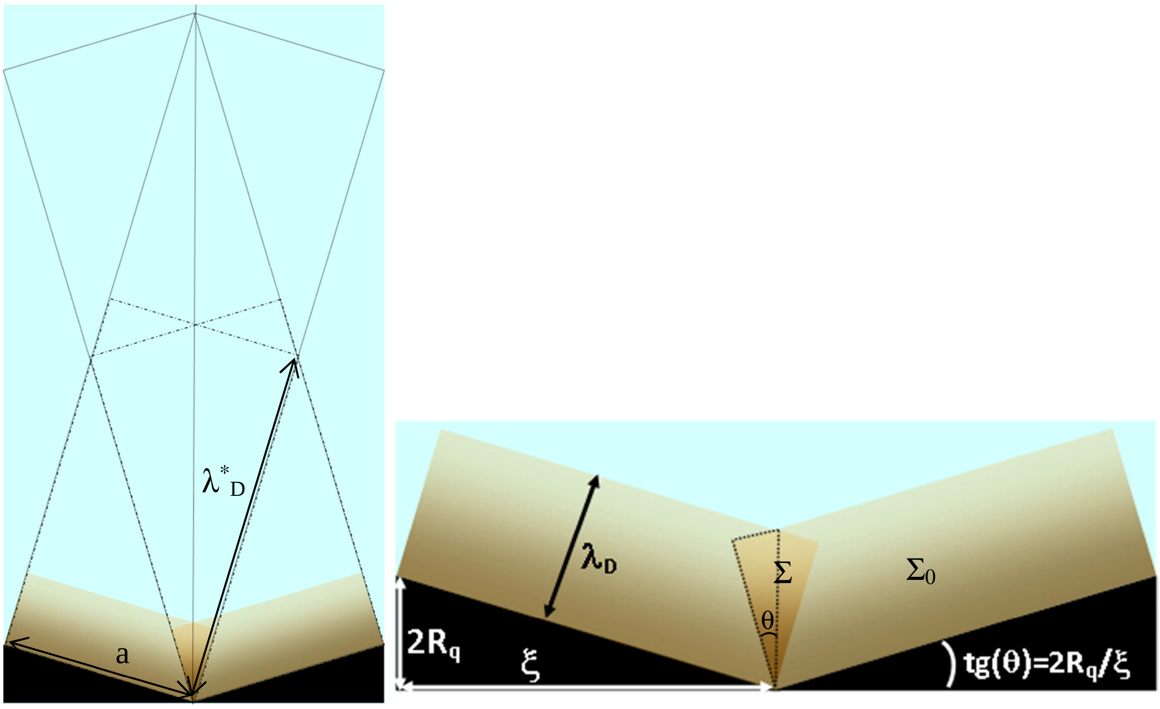

Figure S20. The simplified double layer structure of a surface pore, in the case $\theta\leq 45°$ ($2R_q/\xi\leq 1$). On the left, it can be seen that the shape of the overlapping region is a kite of area Σ for $\lambda_D<\lambda^*_D$. On the right, a magnified view of the double layer structure for $\lambda_D<\lambda^*_D$.

Case I, $\theta \leq 45°$ ($2R_q/\xi \leq 1$) and $\lambda_D \leq \lambda^*_D$

From Fig. S19-left it follows that:

$$\lambda_D^* \tan\theta = \frac{1}{2}\xi\sqrt{1+\left(\frac{2R_q}{\xi}\right)^2}$$

being $a = \xi\sqrt{1+\left(\frac{2R_q}{\xi}\right)^2}$,

so that:

$$\lambda_D^* = \frac{\xi\sqrt{1+\left(\frac{2R_q}{\xi}\right)^2}}{2\left(\frac{2R_q}{\xi}\right)}, \qquad \text{(S5)}$$

In the case of $2R_q/\xi << 1$, the condition $\lambda_D \leq \lambda^*_D$ holds for λ_D up to several times larger than ξ.

Σ is twice the area of the right triangle highlighted by the dotted line in Fig. S20-right:

$$\Sigma = \lambda_D^2\left(\frac{2R_q}{\xi}\right), \qquad \text{(S6)}$$

Σ_0 is twice the area of the double layer of each pore wall $a\lambda_D$ minus the area Σ common overlap region. It follows that:

$$\Sigma_0 = 2\lambda_D\xi\sqrt{1+\left(\frac{2R_q}{\xi}\right)^2} - \lambda_D^2\left(\frac{2R_q}{\xi}\right), \qquad \text{(S7)}$$

Eventually:

$$\gamma = \frac{\left(\frac{\lambda_D}{\xi}\right)\left(\frac{2R_q}{\xi}\right)}{2\sqrt{1+\left(\frac{2R_q}{\xi}\right)^2} - \left(\frac{\lambda_D}{\xi}\right)\left(\frac{2R_q}{\xi}\right)}, \qquad \text{(S8)}$$

At $\lambda_D = \lambda^*_D$, $\gamma = 1/3$.

*Case II, θ>45° (2R_q/ξ>1) and λ_D ≤λ*_D*

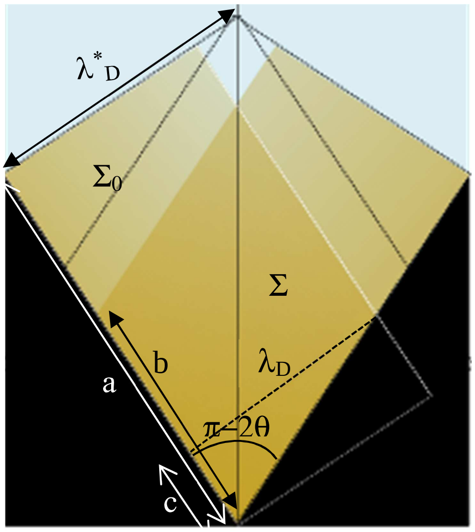

Figure S21. The simplified double layer structure of a surface pore, in the case $\theta>45°$ ($2R_q/\xi>1$). The overlapping region is a rhombus for $\lambda_D<\lambda^*_D$.

$$\lambda_D^* = \frac{\xi\sqrt{1+\left(\frac{2R_q}{\xi}\right)^2}}{\left(\frac{2R_q}{\xi}\right)}$$

In the case of $2R_q/\xi>1$, the condition $\lambda_D \leq \lambda^*_D$ holds for λ_D up to about 1.5ξ.

$$\Sigma = b\lambda_D,$$

$$b = \frac{\lambda_D}{\sin(\pi-2\theta)} = \lambda_D/\sin(2\theta) = \lambda_D/2\sin(\theta)\cos(\theta) = \frac{\lambda_D}{2\tan\theta}[1+(\tan\theta)^2],$$

therefore:

$$\Sigma = \frac{\lambda_D^2}{2}\frac{1+\left(\frac{2R_q}{\xi}\right)^2}{\left(\frac{2R_q}{\xi}\right)}.$$

$$\Sigma_0 = 2a\lambda_D - 2\left(\frac{\lambda_D c}{2}\right) - \Sigma,$$

where $c = \frac{\lambda_D}{\tan(\pi-2\theta)}$ and $\tan(\pi-2\theta) = \frac{2\tan\theta}{(\tan\theta)^2-1}$.

It follows:

$$\Sigma_0 = 2\lambda_D \xi \sqrt{1 + \left(\frac{2R_q}{\xi}\right)^2} - \lambda_D^2 \left(\frac{2R_q}{\xi}\right)$$

as in the case θ≤45°.

$$\gamma = \frac{\left(\frac{\lambda_D}{\xi}\right)\left(\frac{2R_q}{\xi}\right)}{2\sqrt{1+\left(\frac{2R_q}{\xi}\right)^2} - \left(\frac{\lambda_D}{\xi}\right)\left(\frac{2R_q}{\xi}\right)} \frac{1}{2}\left[1 + \frac{1}{\left(\frac{2R_q}{\xi}\right)^2}\right], \tag{S9}$$

At $\lambda_D=\lambda^*_D$, $\gamma>>0.5$.

4. Bibliography

1. Parks G A (1965) The isoelectric points of solid oxides, solid hydroxides, and aqueous hydroxo complex systems. Chemical Reviews 65: 177-198.
2. Gordon E. et al (1999) Metal oxide surfaces and their interactions with aqueous solutions and microbial organisms. Chem. Rev. 99: 77 – 174. DOI: 10.1021/cr980011z.
3. Duval J, Lyklema J, Kleijn J M, van Leeuwen H P (2001) Amphifunctionally Electrified Interfaces: Coupling of Electronic and Ionic Surface-Charging Processes. Langmuir 17: 7573-7581. DOI: 10.1021/la010833i.
4. Duval J, Kleijn J M, Lyklema J, van Leeuwen H P (2002) Double layers at amphifunctionally electrified interfaces in the presence of electrolytes containing specifically adsorbing ions. Journal of Electroanalytical Chemistry 532: 337-352. DOI: 10.1016/S0022-0728.
5. Hsu J P et al (2004) Effect of ionic sizes on the stability ratio of a dispersion of particles with a charge-regulated surface. Journal of colloid and interface science 272: 352 – 357. DOI: 10.1016/j.jcis.2003.10.007.
6. Kallay N, Preocanin T, Kovacevic D, Lützenkirchen J, Chibowski E (2010) Electrostatic Potentials at Solid/Liquid Interfaces. Croat. Chem. Acta 83: 357-370.
7. Hiemstra T, Venema P, Van Riemsdijk W H (1996) Intrinsic proton affinity of reactive surface groups of metal (Hydr)oxides: the bond valence principle. J. of colloid and interface science 184: 680-692. DOI: 10.1006/jcis.1996.0666.
